# Navafenterol (AZD8871) in patients with COPD: a randomized, double-blind, phase I study evaluating safety and pharmacodynamics of single doses of this novel, inhaled, long-acting, dual-pharmacology bronchodilator

**DOI:** 10.1186/s12931-020-01347-7

**Published:** 2020-09-09

**Authors:** Dave Singh, Victor Balaguer, Carol Astbury, Ulrika Wählby-Hamrén, Eulalia Jimenez, Beatriz Seoane, Cristina Villarroel, Alejhandra Lei, Ajay Aggarwal, Ioannis Psallidas

**Affiliations:** 1 The Medicines Evaluation Unit, Centre for Respiratory and Allergy Medicine, University of Manchester, University Hospital of South Manchester NHS Foundation Trust, M23 9QZ, Manchester, UK; 2 Research and Early Development, Respiratory, Inflammation and Autoimmune, BioPharmaceuticals R&D, AstraZeneca, Barcelona, Spain; 3 Clinical Pharmacology and Quantitative Pharmacology, Clinical Pharmacology & Safety Sciences, R&D, AstraZeneca, Gothenburg, Sweden; 4 Clinical Pharmacology and Quantitative Pharmacology, Clinical Pharmacology & Safety Sciences, R&D, AstraZeneca, Barcelona, Spain; 5 Biometrics and Information Sciences, Late-Stage Development, BioPharmaceuticals R&D, AstraZeneca, Barcelona, Spain; 6 Late-Stage Development, BioPharmaceuticals R&D; AstraZeneca, Barcelona, Spain; 7 Patient Safety RIA, Chief Medical Office, R&D, AstraZeneca, Barcelona, Spain; 8 Research and Early Development, Respiratory, Inflammation and Autoimmune, BioPharmaceuticals R&D, AstraZeneca, Boston, MA USA; 9 Research and Early Development, Respiratory, Inflammation and Autoimmune, BioPharmaceuticals R&D, AstraZeneca, Gothenburg, Sweden

**Keywords:** Bronchodilator, COPD, MABA, Dual-pharmacology muscarinic receptor antagonist β_2_-adrenoceptor agonist, Safety, Pharmacokinetics

## Abstract

**Background:**

Navafenterol (AZD8871) is a dual-pharmacology muscarinic antagonist β_2−_agonist (MABA) molecule in development for the treatment of chronic obstructive pulmonary disease (COPD). The pharmacodynamics, safety and tolerability of single doses of navafenterol were investigated in patients with moderate to severe COPD.

**Methods:**

This was a randomized, five-way complete cross-over study. Patients received single doses of navafenterol 400 μg, navafenterol 1800 μg and placebo (all double-blind) and indacaterol 150 μg and tiotropium 18 μg (both open-label active comparators). The primary pharmacodynamic endpoint was change from baseline in trough forced expiratory volume in 1 s (FEV_1_) on day 2. Safety and tolerability were monitored throughout.

**Results:**

Thirty-eight patients were randomized and 28 (73.7%) completed the study. Navafenterol 400 μg and 1800 μg demonstrated statistically significant improvements vs placebo in change from baseline in trough FEV_1_ (least squares mean [95% confidence interval]: 0.111 [0.059, 0.163] L and 0.210 [0.156, 0.264] L, respectively, both *P* < .0001). The changes were significantly greater with navafenterol 1800 μg vs the active comparators (least squares mean treatment difference: 0.065–0.069 L, both *P* < .05). The frequency of treatment-emergent adverse events was similar for placebo and the active comparators (range 34.4–37.5%), slightly higher for navafenterol 400 μg (52.9%), and lowest for navafenterol 1800 μg (22.6%).

**Conclusions:**

Both doses of navafenterol demonstrated sustained bronchodilation over 24 h. Navafenterol was well tolerated and no safety concerns were raised.

**Trial registry:**

ClinicalTrials.gov; No.: NCT02573155; URL: www.clinicaltrials.gov. Registered 9th October, 2015.

## Background

Inhaled long-acting bronchodilators are integral in the pharmacological management of chronic obstructive pulmonary disease (COPD). Whilst bronchodilator monotherapy is recommended for treatment initiation in many patients with COPD [1], there is considerable evidence that the combination of a long-acting muscarinic receptor antagonist (LAMA) with a long-acting β_2_-agonist (LABA) offers additional benefits over monotherapy [2, 3]. The Global Initiative for Chronic Obstructive Lung Disease (GOLD) report recommends a combination of a LAMA and LABA for treatment escalation, or initiation, in patients with greater symptom burden or exacerbation risk [1].

Co-formulation of fixed-dose combinations (FDCs), including LAMA/LABA combinations, is technically challenging [4]. The development of dual-pharmacology muscarinic antagonist β_2−_agonist (MABA) molecules offers a new approach to the treatment of COPD [4]. These molecules combine two mechanisms of action within a single entity, offering potential advantages such as the delivery of a fixed ratio of LAMA/LABA activity into each lung region, simplification of technical and clinical development, and the potential for additive or synergistic bronchodilation over either entity alone [4, 5]. The use of MABAs also creates a platform for the inclusion of another drug, such as an inhaled corticosteroid (ICS) or another anti-inflammatory agent [4]. Navafenterol (AZD8871, formerly LAS191351) is one of the few MABAs in clinical development for the treatment of COPD. Its pharmacological profile has been extensively studied in preclinical investigations in vitro and in vivo, and these investigations have confirmed its dual action at β_2_- and muscarinic receptors [6].

A study of navafenterol was conducted in two parts: a first-in-human single ascending-dose study in patients with mild, persistent asthma (Part 1) and a five-way crossover, single-dose study in patients with moderate to severe COPD (Part 2). In Part 1, single ascending doses of navafenterol 50, 200, 400, 900, 1800, and 2100 μg were well tolerated and doses ≥200 μg produced clinically meaningful, sustained bronchodilation [7]. Here, we report data from Part 2 of this study; the primary objective was to assess the pharmacodynamics, safety, and tolerability of single doses of navafenterol in patients with moderate to severe COPD, with exploratory comparisons vs placebo and the active comparators indacaterol (a LABA) and tiotropium (a LAMA).

## Methods

### Study design

This was a randomized, double-blind, five-way complete crossover, placebo and active-controlled single-dose study (NCT02573155). A subset of patients participated in a pharmacokinetic (PK) sub-study, the methods and results of which are reported in Additional file 1. The study was conducted at a single site in the UK (Medicines Evaluation Unit, Manchester). The study protocol was approved by an Independent Ethics Committee (NRES Committee – Cambridgeshire and Hertfordshire, Health Research Authority, Nottingham, UK; Reference No 15/EE/0329; see Additional file 1) and the UK Medicines and Healthcare Products Regulatory Agency. The informed consent form was also reviewed by the Independent Ethics Committee. The study was performed in accordance with the Declaration of Helsinki and the International Conference on Harmonization/Good Clinical Practice guidelines. All patients provided written, informed consent before enrolment in the study. The first patient was randomized on April 25, 2016; the last patient visit was August 22, 2016.

**Fig. 1:**
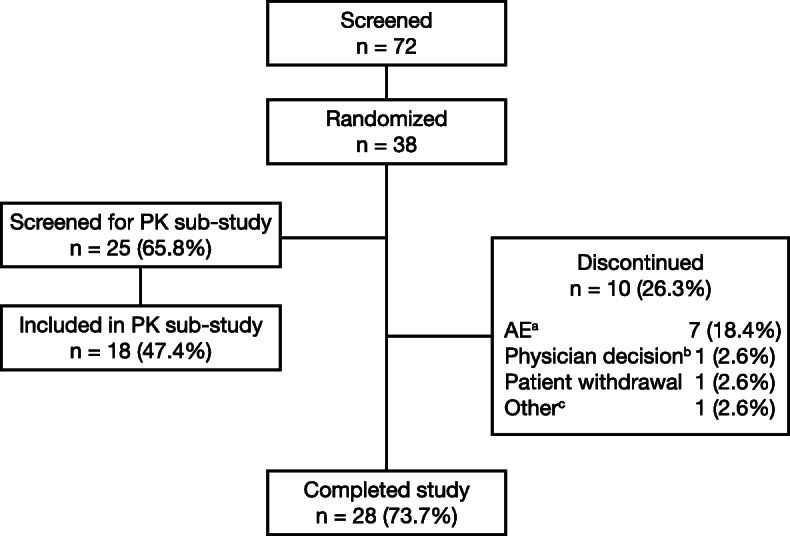
Patient disposition and flow. AE = adverse event; PK = pharmacokinetic. ^a^Five patients discontinued due to treatment emergent AEs leading to study drug discontinuation, 1 patient withdrew due to a treatment emergent AE during the washout period, and 1 withdrew due to a non-treatment-emergent AE; ^b^patient had a positive drug screen test for cocaine; ^c^patient did not meet stability/variability criteria

**Fig. 2:**
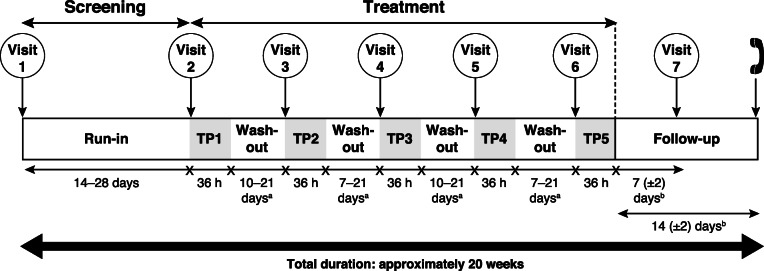
Study design. TP = treatment period. ^a^The figure shows an example of the washout timings for one patient. The washout period was 10 to 21 days following navafenterol or placebo, and 7 to 21 days following the active comparators indacaterol and tiotropium. Therefore the length of each washout period depended on the patient’s randomization sequence. One exception per patient of up to 28 days was considered acceptable; ^b^days following administration of last dose

Following a screening evaluation (Fig. 1) and run-in period of 14 to 28 days, patients were assigned to one of 10 treatment sequences, each with a 1:1:1:1:1 randomization ratio, according to a William’s design for crossover studies (Fig. 2). There were five treatment periods, with patients remaining on-site for 36 h post-dose in each treatment period. The treatments administered were navafenterol 400 μg, navafenterol 1800 μg, and placebo (all double blind, each administered via a variant of the Genuair™/Pressair®^a^ dry-powder inhaler [DPI] adapted internally to deliver a single dose of inhalation powder) and indacaterol 150 μg and tiotropium 18 μg (both open-label, delivered by Onbrez Breezhaler® and HandiHaler® DPIs, respectively). A single dose was administered at the same time (±1 h) on day 1 of each treatment period, between 7 am and 10 am. There was a washout period of 10 to 21 days following navafenterol or placebo treatment and 7 to 21 days following indacaterol or tiotropium treatment.

Patients were withdrawn from their usual COPD therapy after signing the informed consent form but maintained on their usual ICS dose; those receiving an ICS/LABA FDC were switched to ICS monotherapy. Salbutamol was provided as reliever medication for the duration of the study.

A follow-up visit was performed 7 (±2) days after the last dose or after discontinuation. Patients were contacted by telephone 14 (±2) days after the last dose, to record any adverse events (AEs).

### Patients

Male and female (of non-child-bearing potential) patients aged ≥40 years with a clinical diagnosis of stable moderate to severe COPD according to GOLD 2015 criteria [8] were eligible for inclusion. Other inclusion criteria included: postbronchodilator forced expiratory volume in 1 s (FEV_1_) < 80% and ≥ 30% predicted normal and FEV_1_/forced vital capacity (FVC) ratio < 70%; body mass index < 40 kg/m^2^, current or ex-smoker with a smoking history of ≥10 pack-years, no evidence of clinically significant respiratory and/or cardiovascular conditions or laboratory abnormalities and no history of thoracic surgery. Other inclusion and exclusion criteria and study restrictions are reported in Additional file 1.

### Assessments

#### Pharmacodynamics

The primary pharmacodynamic endpoint was change from baseline in trough FEV_1_. Trough FEV_1_ was the mean of the FEV_1_ values obtained at 23 h and 24 h after study drug administration. Secondary endpoints included change from baseline in normalized FEV_1_ area under the curve from 0 to 6 h (AUC_0–6_), 0–12 h (AUC_0–12_), 12–24 h (AUC_12–24_), and 0–24 h (AUC_0–24_) post-dose, change from baseline in FEV_1_ at each scheduled timepoint post-dose, number and percentage of patients achieving ≥100 mL change from baseline in FEV_1_ (minimal clinically important difference) during the 6 h post-dose, change from baseline in and time to peak FEV_1_ on day 1, and change from baseline in trough FVC on day 2.

Details of the timing and measurement standards for FEV_1_ and FVC assessments are provided in Additional file 1.

#### Safety and tolerability

AEs were collected from consent until the telephone follow-up. Treatment-emergent AEs (TEAEs) were defined as AEs that appeared or worsened after the first dose of study drug and within 14 days following the last dose. Other safety assessments included physical examination, blood pressure, clinical laboratory assessments (blood chemistry, hematology, urinalysis, and blood potassium and glucose [both measured via i-STAT]), and 12-lead electrocardiography (see Additional file 1).

### Statistical analysis

There was no formal sample size calculation. It was considered that a sample size of 30 patients would be sufficient to meet the objectives of the study. Approximately 40 patients were randomized to account for an approximate 25% dropout rate.

Pharmacodynamic variables were analyzed in the per protocol population, defined as all randomized patients who satisfied the main inclusion/exclusion criteria, completed at least one treatment period, and had no major protocol violations. All primary and secondary pharmacodynamic variables, with the exception of time to peak FEV_1_, were analyzed by an analysis of covariance model for crossover designs. All statistical comparisons used 2-sided hypothesis tests, and the significance level was set at .05 without multiplicity adjustment. Further details are in Additional file 1.

Safety outcomes were analyzed descriptively in the safety population (all randomized patients who received at least one dose of the study drug).

SAS version 9.2 or later (SAS Institute, Inc., Cary, NC) was used for all statistical analyses.

## Results

### Patient demographics and baseline characteristics

A total of 72 patients were screened; of these, 38 were randomized and 28 (73.7%) completed the study (Fig. 1). Patient demographics and baseline characteristics for the safety population are presented in Table 1. Twenty-two (57.9%) patients had moderate airflow limitation and 16 (42.1%) had severe airflow limitation. Mean (standard deviation [SD]) FEV_1_ absolute reversibility was 217 (124) mL. Mean percentage (SD) bronchodilator reversibility was 19.9% (13.7%). Nineteen patients (50.0%) demonstrated reversibility (postbronchodilator increase in FEV_1_ ≥ 12% and ≥ 200 mL).

**Table 1:** Patient Demographics and Baseline Characteristics (Safety Population)

Baseline Characteristic	Total
Number of patients in safety population	38
Age, years	65.6 (6.4)
Male, *n* (%)	22 (57.9)
White, *n* (%)	38 (100)
Body mass index, kg/m^2^	27.7 (3.5)
Smoking status, *n* (%)	
Current	14 (36.8)
Former	24 (63.2)
Smoking consumption, pack-years	45.4 (26.8)
COPD severity, *n* (%)	
Moderate	22 (57.9)
Severe	16 (42.1)
Duration of COPD, years	9.5 (5.9)
Number of COPD exacerbations in previous 12 months, *n* (%)	
1	8 (21.1)
2	6 (15.8)
3	2 (5.3)
Postbronchodilator % predicted FEV_1_	52.0 (12.5)
Postbronchodilator FEV_1_/FVC	45.8 (10.2)
Bronchial reversibility, %	19.89 (13.72)
FEV_1_ absolute reversibility, L	0.217 (0.124)

*COPD* chronic obstructive pulmonary disease, *FEV*_*1*_ forced expiratory volume in 1 s, *FVC* forced vital capacity

Data are mean (standard deviation) unless otherwise stated

### Pharmacodynamics

**Fig. 3:**
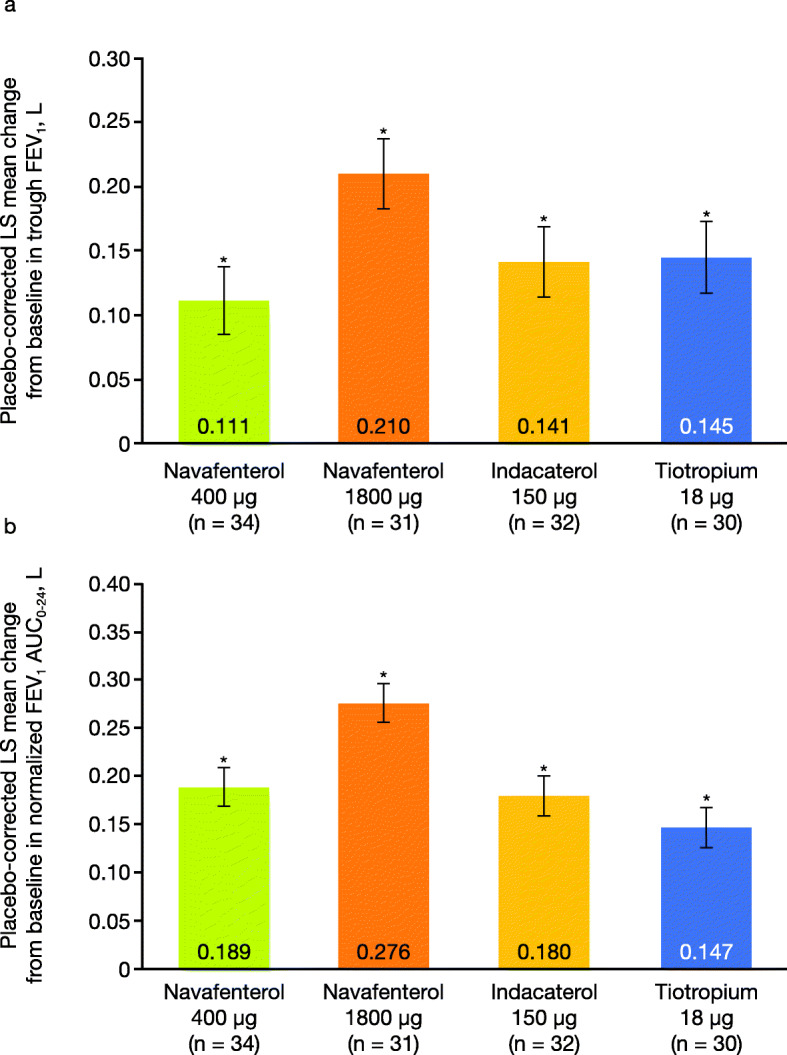
Placebo-corrected mean change from baseline in **a** trough FEV_1_ at day 2 and **b** normalized FEV_1_ AUC_0–24_ (per protocol population). Data are LS means ± standard error. AUC_0–24_ = area under the curve from 0 to 24 h post-dose; FEV_1_ = forced expiratory volume in 1 s; LS = least squares. *P < .0001 vs placebo

All active treatments showed statistically significant improvements vs placebo in change from baseline in trough FEV_1_ (Fig. 3a; Table 2; least squares [LS] mean treatment difference: navafenterol 400 μg, 0.111 L; navafenterol 1800 μg, 0.210 L; indacaterol 150 μg, 0.141 L; tiotropium 18 μg, 0.145 L; all *P* < .0001). The magnitude of change in trough FEV_1_ was greater with navafenterol 1800 μg compared with both active comparators (LS mean treatment difference 0.065–0.069 L, both *P* < .05). A similar pattern of results with a greater effect of navafenterol 1800 μg compared with both active comparators was observed for change from baseline in normalized FEV_1_ AUC_0–24_, AUC_0–12_, and AUC_12–24_. Navafenterol 400 μg also achieved statistically significant improvements vs tiotropium for AUC_0–24_ and AUC_0–12_ (Fig. 3b; Table 2).

**Table 2:** Primary and Secondary Pharmacodynamic Endpoints (Per Protocol Population)

Change From Baseline	Placebo (*n* = 32)^a^	navafenterol 400 μg (*n* = 34)	navafenterol 1800 μg (*n* = 31)	Indacaterol 150 μg (*n* = 32)	Tiotropium 18 μg (*n* = 30)
Trough FEV_1_ on day 2, L (primary endpoint)					
Trough FEV_1_ value	−0.040 (− 0.093, 0.013)	0.071 (0.019, 0.123)	0.170 (0.166, 0.224)	0.101 (0.048, 0.154)	0.105 (0.051, 0.159)
Difference vs placebo		0.111 (0.059, 0.163)	0.210 (0.156, 0.264)	0.141 (0.087, 0.195)	0.145 (0.090, 0.200)
*P*-value		< .0001	< .0001	< .0001	< .0001
Difference vs indacaterol		−0.030 (−0.083, 0.023)	0.0689 (0.015, 0.123)		
*P*-value		.2600	.0126		
Difference vs tiotropium		−0.034 (−0.088, 0.020)	0.0651 (0.011, 0.120)		
*P*-value		.2141	.0197		
Peak FEV_1_ on day 1, L					
Peak FEV_1_ value	0.121 (0.044, 0.198)	0.339 (0.264, 0.413)	0.406 (0.328, 0.483)	0.348 (0.271, 0.424)	0.260 (0.181, 0.340)
Difference vs placebo		0.218 (0.122, 0.314)	0.285 (0.186, 0.384)	0.227 (0.128, 0.326)	0.139 (0.039, 0.240)
*P*-value		< .0001	< .0001	< .0001	.0069
Difference vs indacaterol		−0.009 (−0.106, 0.088)	0.058 (−0.041, 0.157)		
*P*-value		.8556	.2476		
Difference vs tiotropium		0.079 (−0.020, 0.178)	0.146 (0.045, 0.246)		
*P*-value		.1180	.0048		
Normalized AUC_0–24_ of FEV_1_, L					
Normalized FEV_1_ AUC_0–24_ value	−0.021 (−0.075, 0.032)	0.168 (0.115, 0.221)	0.255 (0.201, 0.309)	0.159 (0.105, 0.212)	0.126 (0.072, 0.180)
Difference vs placebo		0.189 (0.150, 0.228)	0.276 (0.236, 0.316)	0.180 (0.139, 0.220)	0.147 (0.106, 0.188)
*P*-value		< .0001	< .0001	< .0001	< .0001
Difference vs indacaterol		0.010 (−0.030, 0.049)	0.096 (0.056, 0.136)		
*P*-value		.6305	< .0001		
Difference vs tiotropium		0.042 (0.002, 0.082)	0.129 (0.088, 0.169)		
*P*-value		.0406	< .0001		
Normalized AUC_0–12_ of FEV_1_, L					
Normalized FEV_1_ AUC_0–12_ value	−0.022 (−0.081, 0.036)	0.233 (0.175, 0.290)	0.301 (0.242, 0.359)	0.192 (0.134, 0.250)	0.156 (0.096, 0.215)
Difference vs placebo		0.255 (0.211, 0.299)	0.323 (0.278, 0.368)	0.214 (0.169, 0.260)	0.178 (0.132, 0.224)
*P*-value		< .0001	< .0001	< .0001	< .0001
Difference vs indacaterol		0.041 (−0.004, 0.085)	0.109 (0.063, 0.154)		
*P*-value		.0732	< .0001		
Difference vs tiotropium		0.077 (0.032, 0.123)	0.145 (0.099, 0.191)		
*P*-value		.0010	< .0001		
Normalized AUC_12–24_ of FEV_1_, L					
Normalized FEV_1_ AUC_12–24_ value	−0.029 (−0.083, 0.025)	0.104 (0.052, 0.156)	0.212 (0.158, 0.266)	0.128 (0.075, 0.181)	0.100 (0.045, 0.154)
Difference vs placebo		0.133 (0.085, 0.180)	0.241 (0.192, 0.289)	0.157 (0.108, 0.205)	0.129 (0.079, 0.178)
*P*-value		< .0001	< .0001	< .0001	< .0001
Difference vs indacaterol		−0.024 (−0.071, 0.023)	0.084 (0.037, 0.131)		
*P*-value		.3172	.0007		
Difference vs tiotropium		0.004 (−0.044, 0.052)	0.112 (0.064, 0.160)		
*P*-value		.8591	< .0001		

*AUC*_*0–24*_ area under the curve from 0 to 24 h post-dose, *AUC*_*0–12*_ area under the curve from 0 to 12 h post-dose, *AUC*_*12–24*_ area under the curve from 12 to 24 h post-dose, *FEV*_*1*_ forced expiratory volume in 1 s. Values are least squares means (95% confidence interval).

^a^Two patients from the placebo group were excluded from the normalized FEV_1_ AUC_12–24_ analysis due to missing data. One patient was missing data from 12 to 24 h post-dose and the other from 14 h post-dose. For all other endpoints in this table, all patients exposed to each treatment were included in the analyses

Mean (SD) time to peak FEV_1_ was 3.4 (2.1) h, 3.9 (2.0) h, 3.0 (2.2) h, and 3.3 (2.2) h with navafenterol 400 μg, navafenterol 1800 μg, indacaterol, and tiotropium, respectively. All active treatments showed statistically significant improvements in change from baseline in peak FEV_1_ vs placebo (e-Fig. 1; Table 2); navafenterol 400 μg, 0.218 L; navafenterol 1800 μg, 0.285 L; indacaterol 150 μg, 0.227 L; tiotropium 18 μg, 0.139 L; *P* < .01 for all). Improvements in peak FEV_1_ with navafenterol 1800 μg were significantly greater compared with tiotropium (*P* < .01) but not indacaterol or navafenterol 400 μg. Improvements with navafenterol 400 μg were similar to those of the active comparators.

**Fig. 4:**
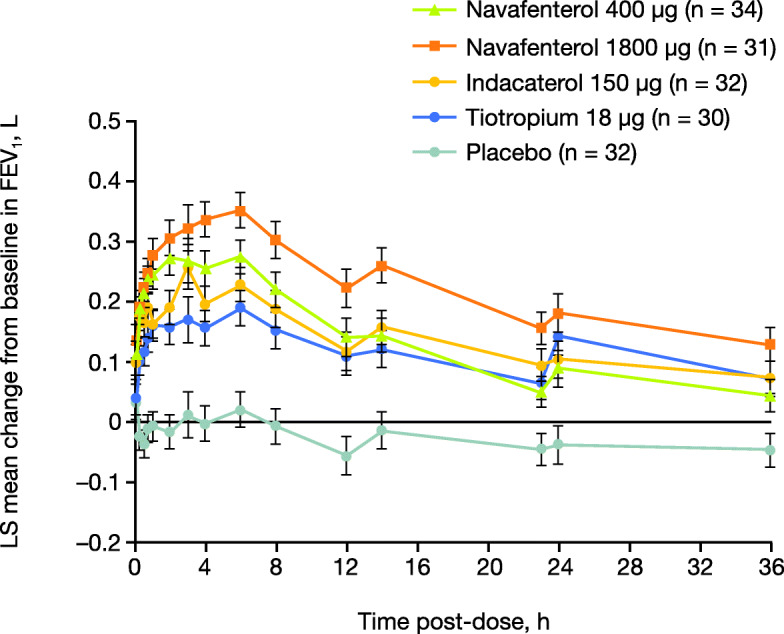
Mean change from baseline in FEV_1_ over 36 h (per protocol population). Data are LS means ± standard error. The number of patients exposed to each treatment differed from the number of non − missing observations for navafenterol 1800 μg (at 45 min and 2 h; both *n* = 30) and placebo (at 12 and 14 h; *n* = 31 and *n* = 30, respectively). FEV_1_ = forced expiratory volume in 1 s; LS = least squares

At all timepoints from 15 min to 36 h post-dose, all active treatments showed statistically significant improvements in change from baseline in FEV_1_ vs placebo (Fig. 4; *P* < .01 for all). Additionally, navafenterol 1800 μg significantly improved change from baseline in FEV_1_ vs placebo at 5 min post-dose (*P* = .0123). FEV_1_ data over time are further described in Additional file 1, along with the proportion of patients achieving ≥100 mL change from baseline in FEV_1_ and trough FVC.

### Safety and tolerability

Overall, 31 (81.6%) patients reported TEAEs (Table 3). The frequency of TEAEs was similar for placebo and the active comparators (range 34.4–37.5%), slightly higher for navafenterol 400 μg (52.9%), and lowest for navafenterol 1800 μg (22.6%). Overall, the most frequently reported TEAEs across all groups were headache (31.6%) and nasopharyngitis (13.2%). Most TEAEs were mild or moderate in intensity and there were no deaths during the study. Only one incidence of headache was considered to be related to the study treatment by the study investigator, which occurred in the tiotropium group. Five (13.2%) patients discontinued treatment due to TEAEs, including two serious AEs (physical assault and fractured C1 vertebra) and three non-serious TEAEs (COPD exacerbation, pneumonia, and headache); none of these TEAEs were assessed as considered related to treatment.

**Table 3:** Frequency of TEAEs Overall and Occurring in ≥2 Patients, by MedDRA^a^ Preferred Term (Safety Population)

TEAE (Preferred Term^a^)	Placebo (*n* = 32)	navafenterol 400 μg (*n* = 34)	navafenterol 1800 μg (*n* = 31)	Indacaterol 150 μg (*n* = 32)	Tiotropium 18 μg (*n* = 30)	All (*N* = 38)
Any event, n (%)	11 (34.4)	18 (52.9)	7 (22.6)	12 (37.5)	11 (36.7)	31 (81.6)
Headache	7 (21.9)	5 (14.7)	3 (9.7)	4 (12.5)	7 (23.3)	12 (31.6)
Nasopharyngitis	1 (3.1)	1 (2.9)	1 (3.2)	2 (6.3)	1 (3.3)	5 (13.2)
COPD	0	2 (5.9)	1 (3.2)	1 (3.1)	0	4 (10.5)
Erythema	1 (3.1)	1 (2.9)	0	1 (3.1)	0	3 (7.9)
Medical device site reaction	1 (3.1)	0	1 (3.2)	1 (3.1)	0	3 (7.9)
Constipation	1 (3.1)	1 (2.9)	1 (3.2)	0	0	2 (5.3)
Nausea	1 (3.1)	1 (2.9)	0	0	0	2 (5.3)
Rhinitis	0	1 (2.9)	0	1 (3.1)	0	2 (5.3)

*COPD* chronic obstructive pulmonary disease, *MedDRA* Medical Dictionary for Regulatory Activities, *TEAE* treatment emergent adverse event.

^a^MedDRA version 18.1

**Fig. 5:**
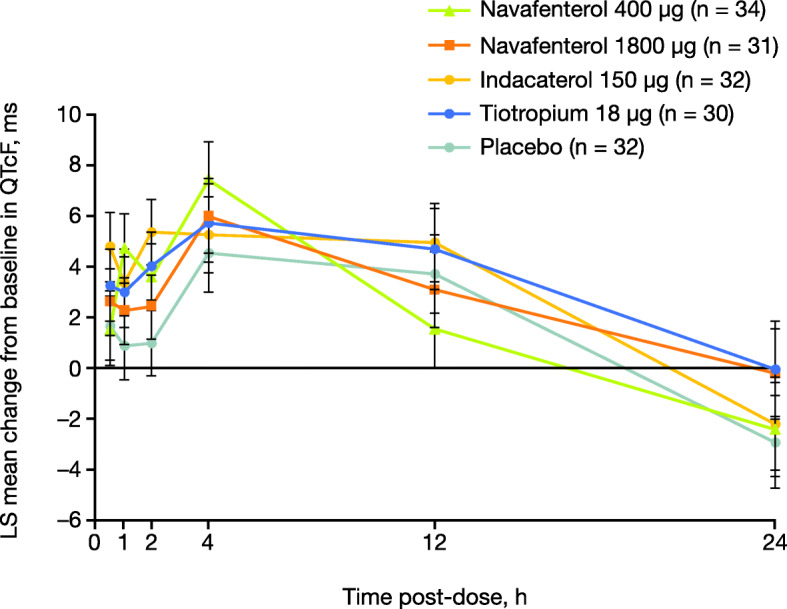
Mean change from baseline in QTcF over time (safety population). Data are LS means ± standard error. QTcF = QT interval corrected for heart rate using the Fridericia formula; LS = least squares

There were no clinically significant changes in clinical laboratory tests, blood glucose and serum potassium concentrations (e-Fig. 2), heart rate, or blood pressure. Small increases in mean change from baseline in QT interval corrected for heart rate using the Fridericia formula (QTcF) were observed in all active treatment groups compared with placebo; the largest increase in LS mean (90% confidence interval) change from baseline in QTcF vs placebo was observed 3 h post-dose with navafenterol 400 μg (3.05 [0.106, 6.00] ms) and navafenterol 1800 μg (5.04 [2.01, 8.06] ms), 2 h post-dose with indacaterol (4.38 [1.84, 6.92] ms), and 36 h post-dose with tiotropium (3.98 [1.27, 6.69] ms). Seven male patients met the criteria for potentially clinically significant QTcF increase, including 6 patients with QTcF values > 450 ms and 1 patient with an increase from baseline > 30 ms; none of these abnormalities were considered clinically significant by the investigator or reported as TEAEs. QTcF values > 450 ms were observed sporadically in all active treatment groups at all timepoints (including baseline) with no clear pattern observed (Fig. 5).

## Discussion

Single doses of navafenterol 400 μg and 1800 μg produced sustained bronchodilation over 24 h in patients with moderate to severe COPD, with significant improvements of 0.111 L and 0.210 L, respectively, in trough FEV_1_ vs placebo. The magnitude of the changes was significantly greater with navafenterol 1800 μg compared with the active comparators, indacaterol (0.069 L) and tiotropium (0.065 L). Both doses of navafenterol significantly improved change from baseline in normalized FEV_1_ AUC_0–24_ vs placebo and tiotropium, with navafenterol 1800 μg also improving this parameter vs indacaterol.

The changes from baseline in trough FEV_1_ with both doses of navafenterol exceeded the minimal clinically important difference of 0.100 L vs placebo [9]. The magnitude of change in trough FEV_1_ vs placebo with navafenterol 400 μg (0.111 L) and 1800 μg (0.210 L) was similar to that observed with single doses of the MABA, batefenterol, in a population of patients with moderate COPD (FEV_1_ 50 to 80% predicted; patients with > 2 exacerbations in the previous 12 months excluded), at doses of 400 μg (0.141 L) and 1200 μg (0.184 L) [10]. Both doses of navafenterol demonstrated a rapid onset of action (within 5–15 min post-dose), with changes from baseline in peak FEV_1_ vs placebo of 0.218 L and 0.285 L with navafenterol 400 μg and 1800 μg, respectively.

One limitation of the study was the inclusion of active comparators as monotherapy rather than combination therapy, meaning that no conclusions can be drawn about the efficacy of AZD8771 compared with LAMA/LABA FDCs. However, the magnitude of the treatment difference between navafenterol 1800 μg and indacaterol 150 μg for trough FEV_1_ (0.069 L) was similar to that observed between the LAMA/LABA FDC DPI glycopyrronium/‌indacaterol 50/110 μg and indacaterol 150 μg (0.07 L) in a 26-week study in patients with moderate to severe COPD [11]. Similarly, the treatment difference between navafenterol 1800 μg and the LAMA tiotropium 18 μg (0.065 L) was similar to that observed between tiotropium/olodaterol 5/5 μg FDC delivered via soft mist inhaler and tiotropium 5 μg (0.050–0.071 L) after 24 weeks’ treatment in patients with moderate to very severe COPD [12]. Whilst it is difficult to draw firm conclusions across studies due to differences in methodologies, these results suggest that navafenterol 1800 μg may provide similar benefits to LAMA/LABA combinations compared with monotherapy. It is important to note that the above-mentioned studies report data following repeat dosing, and the current study was limited by its single-dose design; however, a greater bronchodilatory response for navafenterol has also been achieved with repeat-dosing vs single-dosing [13]. Furthermore, a 4-week study with batefenterol found that greater improvements in FEV_1_ were observed on day 28 compared with day 1 for all doses investigated [14].

The pharmacokinetic data generated for navafenterol are consistent with the rapid onset of action and sustained bronchodilation observed in the pharmacodynamic response. Navafenterol is rapidly absorbed into the bloodstream (median time to maximum concentration 1–2 h) and slowly eliminated from plasma.

Single doses of navafenterol were well tolerated and no safety concerns were identified. The only TEAE occurring in more than two patients in the navafenterol treatment groups was headache; however, its frequency was lower than in the placebo group. Small increases in the duration of QTcF were observed with both doses of navafenterol; however, the upper limit of the 90% confidence interval for the largest QTcF change from baseline vs placebo was < 10 ms. Possible effects of navafenterol on QTcF interval require further evaluation in larger clinical trials with repeat dosing. There were no clinically significant changes in blood glucose and potassium concentrations in the present study.

The proportion of reversible patients in this study was possibly due to the small sample size of the trial. This may have contributed to the effects on lung function observed with navafenterol and the active controls. However, the effects on trough FEV_1_ with navafenterol in this part of the study are in line with the effect size seen in the phase 2a study that only included reversible patients [15]. Since statistical comparisons between navafenterol and the active comparators were made using ANCOVA with no correction for multiple testing, statistical significance should be interpreted with caution, considering the lack of overall control of the type I error. Since this was a first-time-in-human phase I study, additional studies with navafenterol and larger sample sizes will be conducted to elucidate the efficacy of the drug as it progresses through development.

## Conclusion

Both doses of navafenterol demonstrated rapid onset of action (within 5–15 min post-dose) and sustained bronchodilation over 24 h. The bronchodilatory efficacy of navafenterol 1800 μg was greater than that of both indacaterol 150 μg and tiotropium 18 μg. Overall, navafenterol was well tolerated and no safety concerns were raised. These results support the continued clinical development of navafenterol.

^a^Registered trademarks of the AstraZeneca group of companies; for use within the USA as Pressair® and Genuair™ within all other licensed territories.

## Supplementary information

**Additional file 1: Additional information.** The e-Appendices, e-Tables and e-Figures can be found in the Supplemental Materials section of the online article.

## Data Availability

Data underlying the findings described in this manuscript may be obtained in accordance with AstraZeneca’s data sharing policy described at https://astrazenecagrouptrials.pharmacm.com/ST/Submission/Disclosure.

## Abbreviations

AE: Adverse event
AUC_0–∞_: Area under the concentration-time curve from time zero extrapolated to infinity
AUC_0–t_: Area under the concentration-time curve from zero to the last quantifiable measurable concentration
AUC_0–6_: Area under the curve from 0 to 6 h post-dose
AUC_0–12_: Area under the curve from 0 to 12 h post-dose
AUC_0–24_: Area under the curve from 0 to 24 h post-dose
AUC_12–24_: Area under the curve from 12 h to 24 h post-dose
COPD: Chronic obstructive pulmonary disease
DPI: Dry-powder inhaler
FDC: Fixed dose combination
FEV_1_: Forced expiratory volume in 1 s
GOLD: Global Initiative for Chronic Obstructive Lung Disease
FVC: Forced vital capacity
ICS: Inhaled corticosteroid
LABA: Long-acting β_2_-adrenoceptor agonist
LAMA: Long-acting muscarinic receptor antagonist
LS: Least squares
MABA: Muscarinic receptor antagonist and β_2_-adrenoceptor agonist
PK: Pharmacokinetic
QTcF: QT interval corrected for heart rate using the Fridericia formula
SD: Standard deviation
TEAE: Treatment-emergent adverse event
