# Supplementary material for: Navafenterol (AZD8871) in patients with COPD: a randomized, double-blind, phase I study evaluating safety and pharmacodynamics of single doses of this novel, inhaled, long-acting, dual-pharmacology bronchodilator
Source: Respir Res. 2020 Sep 9;21(Suppl 1):102. doi: 10.1186/s12931-020-01347-7 (PMC7487995; doi:10.1186/s12931-020-01347-7)

**Online Supplement**

# e-Appendix 1. Methods

## Study Design

**Ethics committee:** National Research Ethics Service Committee – Cambridgeshire and Hertfordshire Health Research Authority, The Old Chapel, Royal Standard Place, Nottingham, NG1 6FS, UK.

**Blinding and unblinding:** Matching placebo of navafenterol (AZD8871) had identical external appearance (same variant of the Genuair™/Pressair^®a^ device) with the same composition, except for the active ingredient. Indacaterol and tiotropium treatments were open label. Each investigator was provided with a set of sealed emergency envelopes, one per patient and period identifying the treatment group. These envelopes were only to be opened in the case of a medical emergency where the appropriate management of the patient required knowledge of the treatment randomisation. Unblinding of treatment codes after database lock was performed by study site personnel. Treatment codes were not broken for the planned analyses of data until all decisions on the evaluability of the data from each individual patient had been made and documented.

**Procedures for randomisation:** At visit 2, patients who met the inclusion / exclusion criteria were assigned to one of the 10 possible treatment sequences, according to a William’s design for crossover studies and using a balanced randomisation ratio per treatment sequence. Thus, 4 patients were assigned to each treatment sequence. Randomisation was performed in blocks and the randomisation code was assigned from a randomisation list prepared from a computerised system at the study site. Patients were randomised strictly sequentially to the lowest available randomisation code.

## Patients

**Inclusion criteria**: Further to the inclusion criteria outlined in the main methods section, other inclusion criteria included: a negative test for hepatitis B surface antigen, hepatitis B core immunoglobulin M, hepatitis C antibody, and human immunodeficiency virus I and II antibodies at screening; ability to perform repeatable pulmonary function testing for forced expiratory volume in 1 second (FEV1) according to American Thoracic Society (ATS)/ European Respiratory Society (ERS) 2005 criteria^1^ at screening; and ability to change current chronic obstructive pulmonary disease (COPD) therapy and discontinue previous prescribed medications after signature of informed consent, as per required washout periods.

**Exclusion criteria**: Exclusion criteria included: current evidence or recent history of any clinically significant and unstable disease (other than COPD) or abnormality that could put the patient at risk or confound the results; history or current diagnosis of asthma; recent history of COPD exacerbation requiring hospitalisation or need for increased COPD maintenance treatments 6 weeks prior to screening or prior to randomisation; daily oxygen therapy > 10 h per day; lower respiratory tract infection, upper respiratory tract infection requiring antibiotics within 6 weeks prior to screening or prior to randomisation, or use of systemic steroids for respiratory reasons within 6 weeks prior to screening; history of alcohol or drug abuse in past 2 years; QT interval corrected for heart rate using the Fridericia formula (QTcF) > 450 ms for males and > 470 ms for females at screening or prior to randomisation; and history of long QT syndrome.

**Restrictions**: Patients were required to abstain from alcohol 72 h prior to each visit, avoid smoking and caffeine products for 1 h and 8 h, respectively, prior to pulmonary function tests, avoid energy drinks containing taurine or glucuronolactone from 72 h prior to admission until follow-up, and avoid consuming poppy seeds from consent until follow-up. Daily meals were provided during each treatment period. Patients fasted for a minimum of 4 h prior to potassium and glucose (i-STAT measurements) and 8 h prior to other clinical laboratory tests.

## Assessments

**Pharmacodynamic assessments**: FEV_1_ and forced vital capacity (FVC) assessments were performed at screening, 15 and 45 min pre-dose, and at 5, 15, 30, and 45 min and 1, 2, 3, 4, 6, 8, 12, 14, 23, 24 and 36 h post-dose and at follow-up. At each timepoint, three technically acceptable measurements were obtained according to the ATS/ERS acceptability and reproducibility criteria.^1^ For each treatment period, the pre-dose FEV_1_ needed to be within 20% or 200 mL of the pre-dose FEV_1_ of the first treatment period.

**Safety assessments**: Full physical examinations were performed at screening and follow-up and a brief physical examination was performed pre-dose and 36 h post-dose. Blood pressure was determined, in a supine position, at screening, within 1 h pre-dose, at 10 and 30 min, and 1, 2, 3, 4, 8, 12, 24, and 36 h post-dose, and at follow-up. Blood potassium and glucose were determined through i-STAT within 1 h pre-dose and at 30 min and 1, 2, 4, 12, and 24 h post-dose. Other laboratory tests (blood chemistry, haematology, and urinalysis) were performed at screening, 24 h post-dose, and at follow-up, with the exception of the assessment of thyroid-stimulating hormone and thyroxine levels, which was only performed at screening and follow-up, and coagulation, which was only assessed at screening. Twelve-lead digital electrocardiograms (ECGs) were recorded at screening, 1 h pre-dose, 10 and 30 min, and 1, 2, 3, 4, 8, 12, 24, and 36 h post-dose, and at follow-up. Concomitant medication was recorded throughout the study.

**Pharmacokinetic assessments**: Blood samples for pharmacokinetic (PK) analysis were collected in a subset of patients during the placebo and navafenterol treatment periods at pre-dose, and at 5, 15, 30, and 45 min and 1, 2, 3, 4, 6, 8, 12, 24, and 36 h post-dose. PK parameters assessed included maximum plasma drug concentration (C_max_)_,_ time to reach C_max_, area under the concentration-time curve from zero to infinity (AUC_0–∞_) and from zero to the last quantifiable measurable concentration (AUC_0–t_), and terminal elimination half-life (t_½λz_). Plasma concentrations of navafenterol and its metabolite LAS191861 were analysed by means of a fully validated bioanalytical assay based on liquid chromatography-tandem mass spectrometry with a lower limit of quantification of 2 pg/mL. PK parameters were derived using Phoenix^®^ WinNonlin^®^ Version 6.4 for non-compartmental analysis (Certara USA, Inc., Princeton, NJ, USA).

**Statistical analysis**: All primary and secondary pharmacodynamic variables, with the exception of time to peak FEV_1_, were analysed by an analysis of covariance model for crossover designs, including sequence, treatment period, and treatment as fixed effects, patient nested within sequence as a random effect, and the baseline value as covariate. For FEV_1_ and FVC, baseline values were defined as the mean of the 15- and 45-min pre-dose assessments on day 1 of each treatment period. Trough was defined as the mean of the FEV_1_ or FVC values obtained at 23 h and 24 h post-dose and peak as the maximum FEV_1_ value during the first 6 h post-dose. All statistical comparisons used 2-sided hypothesis tests, and the significance level was set at .05 without multiplicity adjustment. The primary treatment comparisons were navafenterol vs placebo. Secondary treatment comparisons were active comparators vs placebo and navafenterol vs the active comparators. Comparisons between treatments were carried out using contrasts on the treatment factor.

PK outcomes were analysed in patients from the per protocol population who participated in the PK sub-study.

^a^Registered trademarks of the AstraZeneca group of companies; for use within the USA as Pressair^®^ and Genuair™ within all other licensed territories

# e-Appendix 2. Results

## Time to peak FEV_1_

Median time to peak FEV_1_ was 3 h with navafenterol 400 μg, indacaterol, and tiotropium, and 4 h with navafenterol 1800 μg.

## FEV_1_ over time

The magnitude of increase from baseline in FEV_1_ vs placebo over 36 h ranged from 74 to 289 mL with navafenterol 400 µg and 101 to 340 mL with navafenterol 1800 µg. Navafenterol 1800 μg was statistically superior to indacaterol from 30 min to 36 h post-dose, with the exception of 3 h post-dose, and to tiotropium from 5 min to 23 h post-dose and at 36 h post-dose (*P* < .05 for all). Navafenterol 400 μg was statistically superior to indacaterol at 30 min, 1, 2, and 4 h post-dose and to tiotropium from 15 min to 8 h post-dose (*P* < .05 for all).

## Proportion of patients achieving ≥ 100 mL change from baseline in FEV_1_

At each timepoint from 30 min to 6 h post-dose, the proportion of patients achieving a ≥ 100 mL change from baseline in FEV_1_ was greater with both doses of navafenterol compared with indacaterol and tiotropium (e-Table 1).

## Trough FVC

All active treatments significantly improved change from baseline in trough FVC vs placebo (range 185–357 mL, all *P* < .001); navafenterol 1800 µg significantly improved trough FVC vs indacaterol and tiotropium (e-Table 2; both *P* < .05); differences between navafenterol 400 µg and the active comparators were not statistically significant.

## Normalised FEV_1_ area under the curve from 0 to 6 h post-dose (AUC_0–6_)

All active treatments showed statistically significant improvements in the change from baseline in normalised FEV_1_ AUC_0–6_ (least squares mean difference vs placebo [95% confidence interval]: navafenterol 400 µg, 0.258 [0.218, 0.297] L; navafenterol 1800 µg, 0.310 [0.270, 0.350] L; indacaterol, 0.206 [0.165, 0.247] L; tiotropium, 0.159 [0.118, 0.200] L; all *P* < .0001). Navafenterol 400 µg and 1800 µg also resulted in significant improvements in normalised FEV_1_ AUC_0–6_ vs both active comparators (all *P* < .05).

## Pharmacokinetics

Eighteen patients (47.4%) participated in the PK substudy. All had evaluable PK parameters and were included in the PK population. Navafenterol was rapidly absorbed following inhalation (median time to t_max_ 1.0 h and 2.0 h for navafenterol 400 µg and 1800 µg, respectively; e-Fig 3). C_max_ and AUC_0–∞_ values for navafenterol increased with dose and demonstrated moderate-to-high inter-patient variability (geometric mean C_max_ [geometric coefficient of variation (GCV)]: navafenterol 400 µg, 199.3 pg/mL [37.4%]; navafenterol 1800 µg, 810.8 pg/mL [36.6%]; geometric mean AUC_0–∞_ [GCV]: navafenterol 400 µg, 1459 pg.h/mL [39.2%]; navafenterol 1800 µg, 6769 pg.h/mL [44.7%]). The arithmetic mean t_½λz_ for navafenterol ranged from 12.9 to 14.2 h***.*** However t_½λz_ estimates should be interpreted with caution as the λz values were obtained from data spanning less than two half-lives. The metabolite LAS191861 appeared shortly after navafenterol (median time to t_max_ 3.0 h for both navafenterol 400 µg and 1800 µg). The mean metabolite to parent ratio based on AUC_0–∞_ was 0.2 for both navafenterol 400 µg and 1800 µg.

# Reference

1. Miller MR, Hankinson J, Brusasco V, et al. Standardisation of spirometry. *Eur Respir J.* 2005;26(2):319-338.

**e-table 1 ]** Number and Percentage of Patients Achieving ≥ 100 mL Change from Baseline in FEV_1_ at Each Timepoint During 6 h Post-dose (Per Protocol Population)

| Time post-dose | No of patients (%) | | | | |
| --- | --- | --- | --- | --- | --- |
|  | Navafenterol 400 µg (n = 34) | Navafenterol 1800 µg (n = 31)^a^ | Indacaterol 150 µg (n = 32) | Tiotropium 18 µg (n = 30) | Placebo (n = 32) |
| 5 min | 18 (52.9) | 19 (61.3) | 21 (65.6) | 5 (16.7) | 5 (15.6) |
| 15 min | 27 (79.4) | 22 (71.0) | 24 (75.0) | 17 (56.7) | 2 (6.3) |
| 30 min | 30 (88.2) | 25 (80.6) | 22 (68.8) | 20 (66.7) | 1 (3.1) |
| 45 min | 31 (91.2) | 26 (83.9) | 25 (78.1) | 21 (70.0) | 2 (6.3) |
| 1 h | 31 (91.2) | 27 (87.1) | 19 (59.4) | 21 (70.0) | 2 (6.3) |
| 2 h | 32 (94.1) | 26 (83.9) | 21 (65.6) | 21 (70.0) | 4 (12.5) |
| 3 h | 32 (94.1) | 28 (90.3) | 26 (81.3) | 21 (70.0) | 5 (15.6) |
| 4 h | 29 (85.3) | 28 (90.3) | 25 (78.1) | 19 (63.3) | 6 (18.8) |
| 6 h | 31 (91.2) | 29 (93.5) | 26 (81.3) | 22 (73.3) | 8 (25.0) |

FEV_1_ = forced expiratory volume in 1 second.

^a^The number of patients exposed to each treatment differed from the number of non−missing observations for navafenterol 1800 µg at 45 min and 2 h (both n = 30).

**e-table 2 ]** Change From Baseline in Trough FVC (Per Protocol Population)

| Change from Baseline Trough FVC on Day 2, L | Placebo (n = 32) | Navafenterol 400 µg (n = 34) | Navafenterol 1800 µg (n = 31) | Indacaterol 150 µg (n = 32) | Tiotropium 18 µg (n = 30) |
| --- | --- | --- | --- | --- | --- |
| Trough FVC value | −0.910 (−0.203, 0.021) | 0.100 (−0.011, 0.210) | 0.266 (0.153, 0.380) | 0.147 (0.035, 0.259) | 0.095 (−0.020, 0.209) |
| Difference vs placebo |  | 0.191 (0.088, 0.293) | 0.357 (0.252, 0.462) | 0.238 (0.132, 0.343) | 0.185 (0.079, 0.292) |
| *P*-value |  | .0003 | < .0001 | < .0001 | .0008 |
| Difference vs indacaterol |  | −0.047 (−0.151, 0.056) | 0.120 (0.015, 0.224) |  |  |
| *P*-value |  | .3694 | .0258 |  |  |
| Difference vs tiotropium |  | 0.005 (−0.100, 0.110) | 0.172 (0.066, 0.278) |  |  |
| *P*-value |  | .9215 | .0018 |  |  |

FVC = forced vital capacity; n = number of patients exposed to each treatment.
Values are least squares means (95% confidence interval).

e-Figure 1 **–** *Placebo-corrected mean change from baseline in peak FEV_1_ at day 1 (per protocol population). Data are LS means ± standard error. FEV_1_ = forced expiratory volume in 1 second; LS = least squares. ^*^P < .0001 vs placebo; ^†^P < .01 vs placebo.*


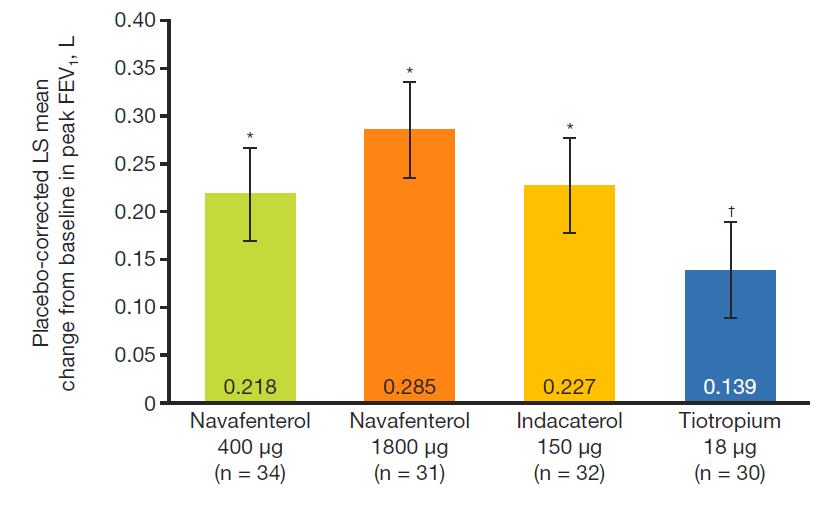


e-Figure 2 – *Mean change from baseline in (a) blood glucose and (b) potassium (safety population). Data are means ± standard deviation.*


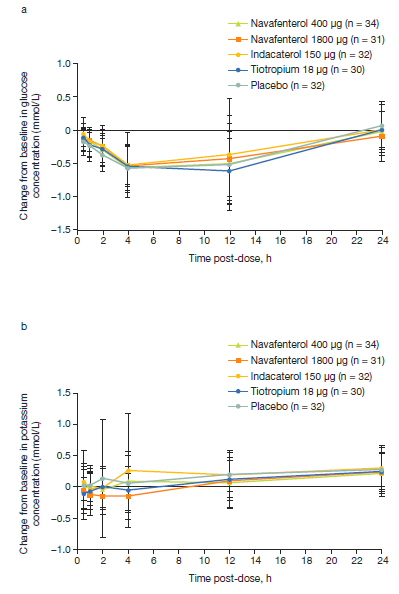


e-Figure 3 – *Geometric mean plasma concentration over time for navafenterol (pharmacokinetic population). Data are shown on a semi-logarithmic scale. For error bars, the geometric mean SD is displayed as exponential (arithmetic mean of the natural log-transformed variable ± arithmetic SD of the natural log-transformed variable). The number of patients exposed to each treatment differed from the number of non−missing observations for navafenterol 400 µg at 12 and 24 h (both n = 17) and 36 h (n = 16), and navafenterol 1800 µg at 45 min (n = 14). SD = standard deviation.*


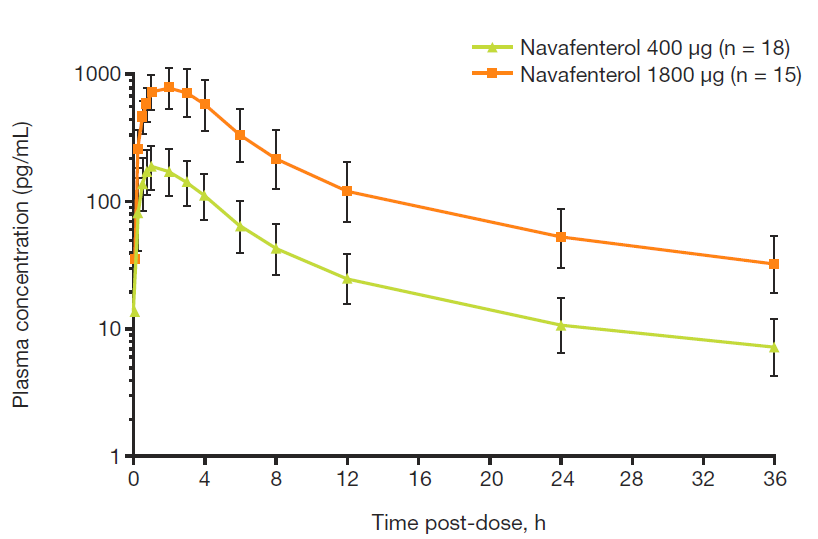

Supplement: Supplementary file 1 — Additional file 1: Additional information. The e-Appendices, e-Tables and e-Figures can be found in the Supplemental Materials section of the online article. [file 12931_2020_1347_MOESM1_ESM.docx]
